# Senescent fibroblasts in aging and pulmonary fibrosis

**DOI:** 10.3389/fragi.2026.1816071

**Published:** 2026-06-11

**Authors:** Sara B. Palega, Aiwei Y. Borengasser, Yan Y. Sanders

**Affiliations:** 1 Department of Biomedical and Translational Sciences, Eastern Virginia Medical School, Macon and Joan Brock Virginia Health Sciences at Old Dominion University, Norfolk, VA, United States; 2 Department of Natural Science, Pulaski Technical College, University of Arkansas, Norfolk, AR, United States

**Keywords:** aging, fibroblasts, lung, pulmonary fibrosis, senescence

## Abstract

Aging is a major risk factor for many chronic lung diseases, including Idiopathic Pulmonary Fibrosis (IPF), a fatal and incurable disease characterized by progressive fibrotic remodeling. Age-associated structural alterations, impaired regenerative capacity, and dysregulated cellular signaling collectively create a pro-fibrotic microenvironment. A central driver of this pathological shift is the accumulation of senescent cells, which undergo irreversible growth arrest and develop a robust pro-inflammatory senescence-associated secretory phenotype (SASP). Emerging evidence identifies senescent lung fibroblasts as critical mediators of IPF pathogenesis. These cells promote excessive extracellular matrix deposition, myofibroblast differentiation, and tissue stiffening, while simultaneously impairing epithelial regeneration. Together, these effects create self-reinforcing feedback loops that perpetuate fibrotic remodeling and disease progression. To therapeutically target this process, strategies to use senolytics and senomorphics have been developed to eliminate senescent fibroblasts or attenuate their pathogenic secretory programs. However, significant translational challenges remain, including senescent cell heterogeneity, the lack of definitive and cell-specific biomarkers, and the need for targeted delivery approaches to enhance precision and minimize off-target effects. In this review, we delineate the mechanisms by which cellular senescence reprograms fibroblast function and disrupts normal lung repair to drive fibrosis, evaluate emerging therapeutic strategies, and discuss the key obstacles needed to be addressed to advance senescence-targeted interventions for IPF.

## 1 Introduction

Aging is a significant risk factor for the development of chronic lung diseases, which represent a major and growing source of global morbidity and mortality (Rojas et al., 2015). Among age-associated interstitial lung diseases, Idiopathic Pulmonary Fibrosis (IPF) is one of the most prevalent and lethal (Prasad et al., 2015). IPF is characterized by aberrant wound-healing, leading to excessive extracellular matrix (ECM) deposition, progressive tissue stiffening, and ultimately irreversible loss of lung function (Wilson and Wynn, 2009). IPF remains incurable, current treatment methods mainly focuses on slowing disease progression but do not reverse or resolve fibrosis (Dempsey et al., 2021; Laporta et al., 2018). Consequently, IPF imposes a substantial and escalating economic burden, costing the United States healthcare system billions of dollars annually (Collard et al., 2015).

During physiological aging, the lungs undergo a gradual decline in functional reserve. This process is marked by reduced gas-exchange surface area, increased vulnerability to infection and injury, and a diminished repair capacity (Wang et al., 2024; Suki et al., 2022). Collectively, these changes reflect a broader age-related cellular deterioration that impair the lung’s ability to respond to stress and maintain homeostasis; predisposing the lung to maladaptive repair (Kapetanaki et al., 2013). Depending on the underlying disease context, this can manifest as excessive matrix deposition and fibrosis as in IPF, or matrix degradation and alveolar destruction as seen in emphysema and COPD (Wang et al., 2024; Schuliga et al., 2021a; Joshi, 2024). IPF is rarely diagnosed before age fifty and incidence rises sharply with age, making it critical to understand how the aging microenvironment predisposes the lung to fibrotic disease (Alsomali et al., 2023; Maher et al., 2021).

**FIGURE 1:**
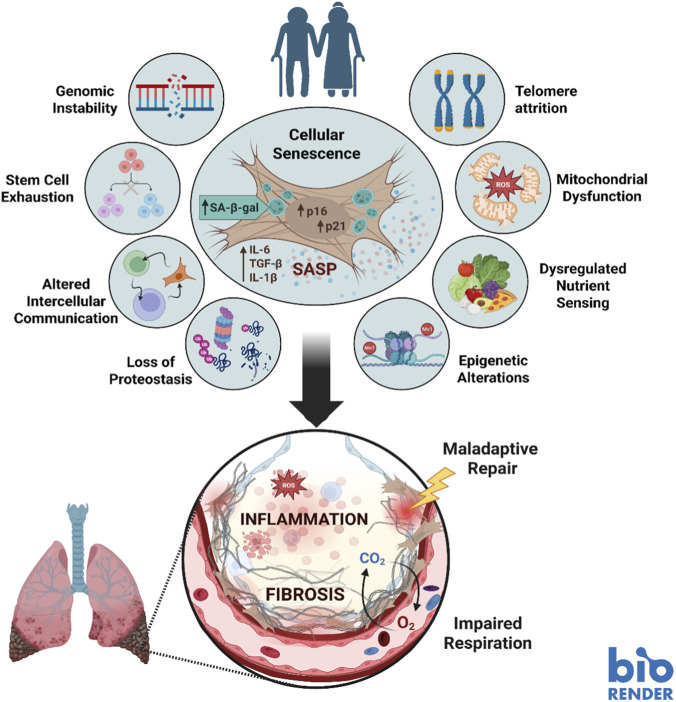
Hallmarks of aging create a proinflammatory and profibrotic microenvironment in aging fibrotic lungs.

At the cellular level, this age-associated decline can be understood through the framework of the hallmarks of aging, a set of interconnected biological processes that progressively deteriorate over time (López-Otín et al., 2013; López-Otín et al., 2023). These hallmarks of aging contribute to the pathogenesis of IPF. For example, stem cell exhaustion erodes the regenerative capacity of the pulmonary epithelium, and cellular senescence drives chronic inflammation and impairs normal tissue repair (Schuliga et al., 2021a; Schafer et al., 2017). Together, these changes disrupt the coordinated wound-healing response, leaving the lung with persistent damage and unresolved inflammatory signaling that predisposes it to chronic disease, such as IPF (Figure 1) (Kapetanaki et al., 2013; Hamsanathan et al., 2019; Waters et al., 2018).

**FIGURE 2:**
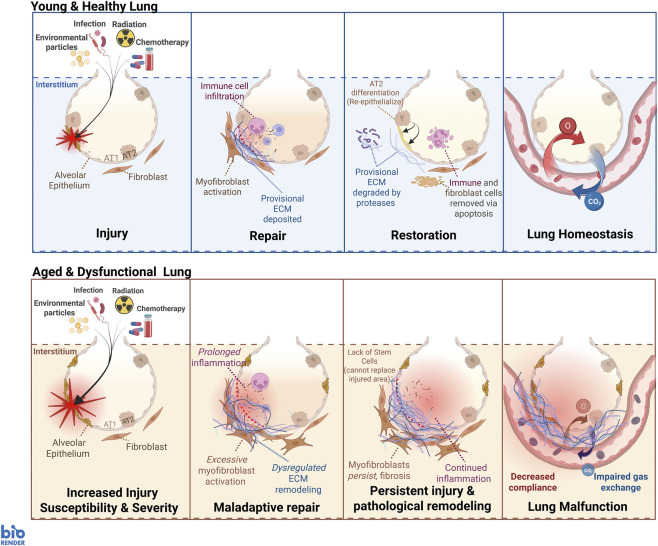
Wound repair in young, healthy lung versus aged, fibrotic lung.

In a young, healthy lung, injury initiates a coordinated repair program. Epithelial cells proliferate to re-establish barrier function, while recruited fibroblasts deposit a provisional extracellular matrix. A subset of these fibroblasts differentiates into myofibroblasts for wound repair, upon resolution, are cleared via apoptosis (Wilson and Wynn, 2009; Schafer et al., 2017; Jun and Lau, 2010). With advancing age, this coordinated response breaks down. Epithelial progenitors become depleted or unresponsive, fibroblasts accumulate in a persistently activated state, and there is uncontrolled deposition of dense, cross-linked scar tissue (Schuliga et al., 2021a; Waters et al., 2018). In the context of IPF, the result is a lung trapped in a state of maladaptive repair, where injury drives fibroblast activation and collagen deposition instead of epithelial regeneration (Figure 2) (Waters et al., 2018; Blokland et al., 2020). It is to note that other age-related lung diseases, such as COPD and emphysema, involve tissue destruction and matrix degradation, but in IPF, the imbalance between repair and resolution locks the lung into progressive scarring (Joshi, 2024). What determines whether an aging lung trends toward fibrosis *versus* degeneration remains an open question, but the failure of normal wound healing is thought to be a major initiating factor in IPF, and it is within this context that senescent fibroblasts emerge as central drivers of disease (Kapetanaki et al., 2013; Jun and Lau, 2010).

## 2 Senescence and aging

Cellular senescence was first described by Hayflick and Moorhead in 1961, who demonstrated that primary human fibroblasts exhibit a finite replicative limit of approximately 50 population doublings before entering a state of irreversible cell-cycle arrest (Hayflick and Moorhead, 1961). This discovery, known as the Hayflick limit, established that normal cells do not divide indefinitely. We now understand that senescence can be triggered by diverse cellular stressors beyond replicative exhaustion, including DNA damage, oxidative stress, and oncogene activation (Campisi and d’Adda Di Fagagna, 2007; Campisi, 2005). Initially, this arrest was viewed primarily as an anti-tumor mechanism, locking damaged cells out of the cell cycle to prevent malignant transformation (Hayflick and Moorhead, 1961). However, a landmark study in 2011 showed that selectively clearing senescent cells from aged mice improved tissue function and delayed the onset of age-related pathologies, establishing a dual role for senescence in which the same program that suppresses tumor formation early in life can become a driver of aging and age-related disease (Baker et al., 2011).

Cellular senescence is defined as a stable, long-term state of cell-cycle arrest, executed primarily through activation of the p16^INK4a^ and/or p21^CIP1^ tumor suppressor pathways in response to genomic insults such as DNA double-strand breaks, telomere erosion, or excessive reactive oxygen species; all of which accumulate with aging and chronic tissue stress (Campisi and d’Adda Di Fagagna, 2007; Chen et al., 2006). Despite their proliferative arrest, senescent cells remain metabolically active and acquire a robust senescence-associated secretory phenotype (SASP), characterized by the production of pro-inflammatory cytokines, chemokines, growth factors, and matrix-remodeling enzymes (Campisi and d’Adda Di Fagagna, 2007; Campisi, 2005; Chen et al., 2006; Lopes-Paciencia et al., 2019). The SASP includes factors such as interleukin-6 (IL-6), IL-1β, IL-8, transforming growth factor-beta (TGF-β), growth differentiation factor 15 (GDF15), and matrix metalloproteinases (Lopes-Paciencia et al., 2019). Morphologically, senescent cells become enlarged and flattened, and display elevated lysosomal activity detectable by senescence-associated β-galactosidase (SA-β-gal) staining (Campisi and d’Adda Di Fagagna, 2007; Campisi, 2005; Dodig et al., 2019). They also become resistant to apoptosis, owing in part to upregulation of anti-apoptotic Bcl-2 family proteins (Yosef et al., 2016). Underlying these phenotypic changes is a broad epigenetic reprogramming that includes silencing of cell cycle–promoting genes, upregulation of SASP components, and alterations in histone modifications that lock cells into the senescent state (Chen et al., 2006; Sanders et al., 2020; Zhu et al., 2026; Wang et al., 2022). Through this cellular reprogramming, senescent cells can become drivers of chronic inflammation, tissue dysfunction, and age-related disease (Zhang et al., 2022).

Identifying senescent cells in tissues is not straightforward. No single marker definitively identifies a senescent cell, largely because senescence is not a uniform state. P21^CIP1^, p16^INK4a^ and SA-β-gal are widely used but must be interpreted with caution, as their expression can vary by cell type, tissue, and initiating stressor (Cohn et al., 2023; Ogrodnik et al., 2024; Ozdemir et al., 2025; Melo-Narváez et al., 2024). SASP composition, surface marker expression, and susceptibility to clearance also differ depending on the cell of origin and the senescence-inducing stimulus, with single-cell studies revealing cell-to-cell variability even within the same population (Melo-Narváez et al., 2024; Maciel-Barón et al., 2016; Wiley et al., 2017). As an example of the heterogeneity issue, eliminating p16^INK4a^-positive cells alleviates the senescent burden and delays age-related pathologies in certain murine models, but this same strategy fails to protect against cellular senescence in models of cigarette-smoke-induced lung disease (Baker et al., 2011; Sundar et al., 2018). This heterogeneity complicates efforts to define senescent populations *in vivo* and represents a major challenge for developing targeted therapies (Ozdemir et al., 2025).

It is important to emphasize that senescence is not inherently pathological. Transient ‘waves’ of senescence plays essential roles in wound healing, where SASP factors promote tissue remodeling and immune cell recruitment; in tumor suppression, where cell-cycle arrest prevents the propagation of damaged cells (Campisi and d’Adda Di Fagagna, 2007; Campisi, 2005; Demaria et al., 2014). Under normal conditions, senescent cells are efficiently cleared by the immune system once their functions are complete. With advancing age, however, immune surveillance declines, and repeated exposure to stressors leads to the gradual accumulation of senescent cells that are not properly cleared (Ovadya et al., 2018). It is thought that this chronic *accumulation*, rather than senescence itself, that becomes pathological (Zhang et al., 2022; Ovadya et al., 2018).

The lungs are particularly vulnerable to senescent cell accumulation. As an organ in constant contact with the external environment, it is repeatedly exposed to various stressors such as pollutants, pathogens, and mechanical stress (Parikh et al., 2019). This high injury burden, combined with an age-related decline in immune clearance, results in a significant ‘senescent cell burden’ within the pulmonary parenchyma (Schuliga et al., 2021a; Hamsanathan et al., 2019; Ovadya et al., 2018; Parikh et al., 2019). In the aging lung, the persistence of senescent cells and their SASP establishes a chronic pro-inflammatory, tissue-damaging milieu that exacerbates diseases such as IPF (Waters et al., 2018; Parikh et al., 2019; Childs et al., 2015). Amplifying this effect, SASP factors induce senescence in neighboring healthy cells through a bystander effect, expanding the senescent cell pool and accelerating pathological tissue remodeling (Acosta et al., 2013; Liao et al., 2014).

Although the fundamental features of senescence, such as cell cycle arrest, SASP production, apoptosis resistance, and metabolic reprogramming, are shared across cell types, the specific stressors that trigger senescence, the composition of the resulting SASP, and the functional consequences for tissue homeostasis vary considerably by cellular context (Cohn et al., 2023; Ogrodnik et al., 2024; Ozdemir et al., 2025; Melo-Narváez et al., 2024; Maciel-Barón et al., 2016). Increasing work has therefore focused on delineating how specific senescent cell subtypes contribute to the pathologies of age-related diseases. As the fibroblasts are the effector cells in lung fibrosis, in the following sections, we focus on examining how the senescence program in the lung fibroblasts drives the pathology of pulmonary fibrosis.

## 3 Fibroblast senescence

In healthy tissue, transient fibroblast senescence contributes to wound repair by secreting factors that promote tissue remodeling and immune cell recruitment (Jun and Lau, 2010). However, in the aging lung, repeated injury and impaired immune clearance lead to the accumulation of senescent fibroblasts, which adopt a persistent and pathogenic phenotype (Schafer et al., 2017; Parikh et al., 2019; Hernandez-Gonzalez et al., 2023). This shift is driven by chronic stressors, such as replicative exhaustion from age and repeated wound-healing cycles, oxidative stress from pollutants or smoke, and persistent DNA damage, which push fibroblasts into senescence (Schafer et al., 2017; Campisi, 2005; Parikh et al., 2019). As mentioned in the previous section, senescence can further propagate through paracrine signaling, whereby the SASP from other senescent cells, such as injured alveolar epithelial cells, induce senescence in neighboring fibroblasts (Liao et al., 2014; Yao et al., 2024).

The progressive accumulation of senescent fibroblasts disrupts normal tissue homeostasis and promotes aberrant repair (Ortiz-Zapater et al., 2022). The functional consequences of this accumulation, however, are not uniform across all age-related lung diseases. For example, in COPD, SASP-derived proteases contribute to matrix degradation and alveolar destruction, whereas in IPF, fibroblasts isolated from patient lungs consistently display senescent features and a pro-fibrotic SASP, and their accumulation correlates with disease severity (Joshi, 2024; Álvarez et al., 2017). Histological studies confirm that senescent fibroblasts are prominent within the fibrotic foci of IPF lungs, and their abundance correlates with disease progression (Álvarez et al., 2017; Rojas et al., 2018; Hall et al., 2016). Fibroblasts isolated from IPF patient tissue display impaired proliferative capacity, elevated SA-β-gal activity, and resistance to apoptosis *ex vivo*, consistent with a senescent phenotype (Álvarez et al., 2017; Yanai et al., 2015). Furthermore, genetic or pharmacological elimination of p16^INK4a^-positive cells in animal models significantly attenuates lung fibrosis, confirming that senescent cells actively drive pathology (Schafer et al., 2017; Baker et al., 2011; Baker et al., 2016). This link between fibroblast senescence and IPF pathogenesis has spurred interest in targeting these cells therapeutically (Ortiz-Zapater et al., 2022; Álvarez et al., 2017; Upagupta et al., 2018). In addition, this pathogenic identity is reinforced through epigenetic reprogramming, including histone modifications that silence cell cycle–promoting genes and upregulate SASP components. In lung fibroblasts global increases in H4K20 trimethylation and decreases in H4K16 acetylation have been linked to genes that are related with senescence-associated resistance to apoptosis or fibrosis (Sanders et al., 2020; Zhu et al., 2026; Sanders et al., 2013). Together, these changes reinforce the senescent phenotype, promoting fibroblast persistence and contributing to disease progression in IPF.

Accumulating senescent fibroblasts fail in their supportive role and instead promote maladaptive repair (Jun and Lau, 2010; Hernandez-Gonzalez et al., 2023; Hernandez-Gonzalez et al., 2021; Lin and Xu, 2020). They are unable to support alveolosphere formation from epithelial progenitor cells, disrupting a critical regenerative pathway (Blokland et al., 2020). Their SASP derails physiological wound healing, reinforcing chronic inflammation and biasing tissue responses toward fibrosis rather than regeneration (Hamsanathan et al., 2019). This secretome is enriched with key profibrotic mediators, including TGF-β, which drives the fibroblast-to-myofibroblast transition by inducing collagen and α-smooth muscle actin (α-SMA) expression (Desmoulière et al., 1993; Frangogiannis, 2020). Collagen is the primary structural protein in fibrotic scar tissue, while α-SMA confers contractile force, enabling myofibroblasts to remodel lung architecture (Molokanova et al., 2018; Tomasek et al., 2005). Senescent fibroblasts also secrete matrix-remodeling enzymes like MMP-2, MMP-9, and MMP-12, which degrade basement membrane and elastin, while Plasminogen Activator Inhibitor-1 (PAI-1) suppresses fibrinolysis. Together, these processes create a disorganized extracellular matrix scaffold that promotes aberrant repair (Aoshiba et al., 2013; Giannandrea and Parks, 2014; Loskutoff and Quigley, 2000; Mavrogonatou et al., 2023). Ultimately, senescent fibroblasts drive fibrosis through excessive and aberrant ECM deposition, overproducing stiff, cross-linked collagen and fibronectin, leading to the progressive scarring and functional decline that characterizes fibrotic disease (Hernandez-Gonzalez et al., 2023; Mavrogonatou et al., 2023).

**FIGURE 3:**
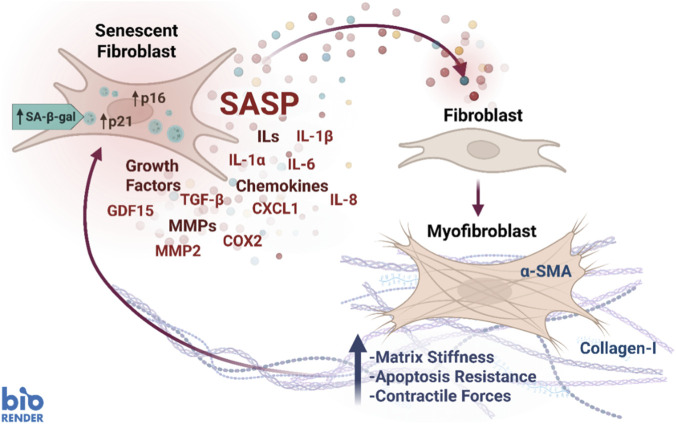
Self-reinforcing feedback loop of lung senescent fibroblasts, fibroblasts, and myofibroblasts (omitting other cells cross talks for simplicity).

This persistence of senescent fibroblasts creates a self-reinforcing cycle in which fibroblasts deposit stiff, cross-linked ECM. This progressive stiffening of the IPF lung further drives fibroblasts activation maintains and myofibroblast differentiation through various signaling pathways, such as YAP/TAZ mechanosensitive signaling (discussed in following section) (Liu et al., 2015; Zhu et al., 2023). At the same time, SASP factors secreted by senescent pulmonary cells sustain chronic inflammation and induce senescence in neighboring cells, expanding the senescent population and amplifying the fibrotic response (Acosta et al., 2013). Collectively, these findings highlight how senescent fibroblasts compromise the normal repair programs and drive self-sustaining fibrotic remodeling, positioning them as a compelling therapeutic target in age-associated fibrotic lung disease (Figure 3). The molecular pathways thought to initiate and sustain fibroblast senescence are discussed in the following section.

## 4 Fibroblast senescence pathways

Although the exact underlying mechanisms by which senescent fibroblasts contribute to IPF remain incompletely understood, several key pathways have been implicated in the senescent reprogramming of the fibroblast, including: NF-κB, cGAS-STING, p38 MAPK, and mTORC1 (Zhu et al., 2026; Moiseeva et al., 2013; Ya and Bayraktutan, 2024; Zhang J. et al., 2023; Xu et al., 2014; Carroll et al., 2017; Salminen et al., 2012; Freund et al., 2011). In the aging lung, fibroblasts encounter several senescence-inducing stimuli relevant to disease, including replicative exhaustion from age and repeated wound-healing cycles, oxidative stress from environmental exposures, and persistent DNA damage from mitochondrial stress (Schafer et al., 2017; Campisi and d’Adda Di Fagagna, 2007). These insults generate damage-associated molecular patterns (DAMPs), including cytosolic DNA from genomic instability and mitochondrial damage, which serve as upstream triggers for the profibrotic related pathways (Huang et al., 2022). It should be noted that most of this work derives from *in vitro* fibroblast models, which lack the full complexity of the fibrotic lung. In addition, some mechanisms have not been directly demonstrated in IPF fibroblasts, and insights from other systems are therefore incorporated (Kolanko et al., 2024).

Among the key pathways, NF-κB serves as a central integration point for several senescence-associated signals. It is activated by multiple stimuli, including reactive oxygen species generated during oxidative stress, cGAS–STING signaling in response to cytosolic DNA, and p38 MAPK activation downstream of persistent DNA damage (Salminen et al., 2012). Once activated, NF-κB drives the expression of inflammatory SASP cytokines including IL-6 and IL-1β (Salminen et al., 2012; Xu et al., 2015). The pathological consequence is sustained inflammatory signaling within the fibroblast population and the surrounding microenvironment. NF-κB also engages in a positive feedback loop with the JAK/STAT pathway: IL-6 activates STAT3, which further amplifies inflammatory gene expression, resulting fibroblasts into a persistent pro-inflammatory state (Yao et al., 2024; Xu et al., 2015). While relevant to lung fibroblasts, NF-κB functions broadly across many cell types and is best understood as a shared inflammatory hub rather than a fibroblast-specific mechanism (Liu et al., 2017).

Another pathway is cGAS-STING, which is triggered by cytosolic DNA that accumulates from genomic instability or mitochondrial damage (Guo et al., 2021). Once activated, this pathway drives NF-κB signaling and type I interferon responses, amplifying the inflammatory SASP program. The downstream consequence is sustained production of pro-inflammatory cytokines that contribute to the chronic inflammatory and tissue damaging environment of the aging lung (Zhang J. et al., 2023; Guo et al., 2021; Schuliga et al., 2021b). Direct evidence for this pathway in fibroblast senescence is limited; most studies have been conducted in epithelial cells, which may have paracrine effects on fibroblasts, and its specific role in IPF fibroblasts remains unclear (Zhang J. et al., 2023; Schuliga et al., 2021b).

Sustained DNA damage also triggers the p38 MAPK pathway (Freund et al., 2011). In senescent lung fibroblasts, p38 stabilizes SASP mRNA transcripts, allowing continuous cytokine production (Freund et al., 2011). More recently, p38 MAPK has been shown to promote histone acetylation at profibrotic gene promoters, sustaining the expression of α-SMA and collagen (Zhu et al., 2026). The pathological consequence is reinforced expression of pro-fibrotic genes alongside persistent SASP output (Zhu et al., 2026; Freund et al., 2011). Like cGAS-STING, p38 signaling feeds into NF-κB, further amplifying the inflammatory program. p38 activity can also be enhanced by mTORC1, connecting it to the metabolic and mechanosensitive regulation (Freund et al., 2011; Herranz et al., 2015).

mTORC1 is triggered in part by the stiff ECM characteristic of the fibrotic lung. Increased matrix rigidity activates YAP/TAZ mechanosensitive signaling, which in turn promotes mTORC1 activity through integrin-mediated pathways (Liu et al., 2015; Han et al., 2025). Once activated, mTORC1 drives a metabolic shift toward glycolysis to fuel the energetic demands of SASP production and promotes resistance to apoptosis, allowing senescent fibroblasts to persist rather than be cleared (Carroll et al., 2017; James et al., 2015; Romero et al., 2016; Laberge et al., 2015). The pathological consequence is sustained senescent cell survival and continued secretory activity within the stiffened fibrotic niche. This metabolic and survival programming is further reinforced by crosstalk with the p38 MAPK pathway (Herranz et al., 2015; Laberge et al., 2015).

**FIGURE 4:**
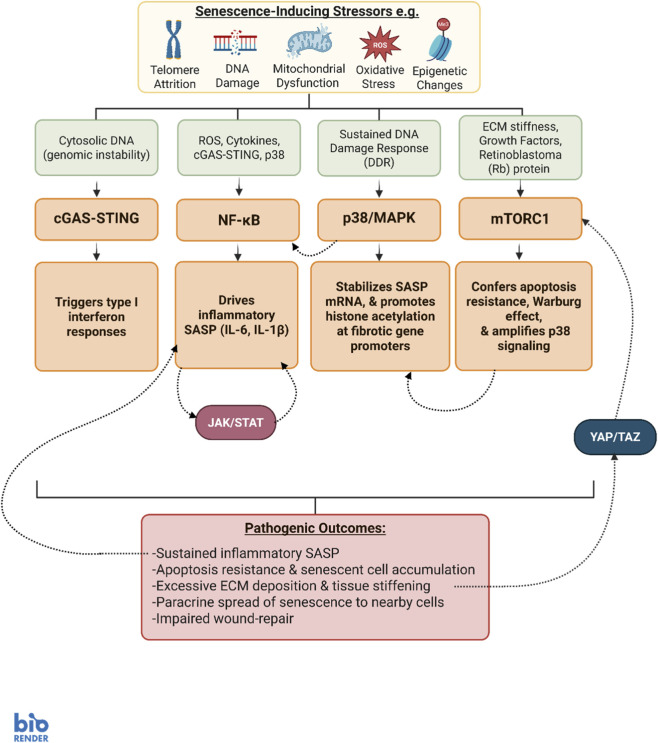
Examples of molecular pathways driving senescence and the pro-fibrotic SASP. Senescence-inducing stressors activate some key signaling pathways that promote inflammatory SASP production, metabolic reprogramming, and apoptosis resistance, reinforced by positive feedback loops that sustain fibrosis. P38 MAPK, NF-κB, and mTORC1 have established roles in lung fibroblast senescence, whereas cGAS-STING has been studied primarily in epithelial senescence and its contribution to fibroblast-driven fibrosis remains unclear.

Together, these pathways form an interconnected signaling network that contributes to the core features of the senescent fibroblast phenotype: cell cycle arrest, SASP production, metabolic reprogramming, apoptosis resistance, and epigenetic remodeling (Figure 4). These highlighted regulators here, NF-κB, mTORC1, p38 MAPK, and cGAS-STING, only represent a subset of a broad and still evolving signaling network in lung fibroblast senescence. Understanding how these pathways drive the senescent phenotype provides a foundation for the therapeutic strategies for this senescence mediated fibrotic disease.

## 5 Targeting fibroblast senescence

Emerging studies have established a role of senescent cells in tissue dysfunction, providing a strong rationale for targeting them as a therapeutic strategy in age-related diseases like IPF (Niedernhofer and Robbins, 2018). Current anti-senescence approaches fall into two broad categories: senolytics, which induce apoptosis in senescent cells; and senomorphics, which suppress SASP without eliminating the cells (Zhang et al., 2022; Niedernhofer and Robbins, 2018; Zhang L. et al., 2023). In fibrotic diseases like IPF, therapeutic efforts have primarily focused on eliminating senescent fibroblasts, which are the principal drivers of pathological extracellular matrix deposition and scar formation (Hernandez-Gonzalez et al., 2023; Lin and Xu, 2020). However, senescent alveolar epithelial and immune cells also contribute to the pro-fibrotic niche through paracrine signaling and inflammatory amplification, so achieving sustained therapeutic benefit likely requires addressing the broader senescent microenvironment rather than targeting one cell type alone (Yao et al., 2024; Su et al., 2021). Due to this complexity, optimizing senolytic and senomorphic interventions remains a critical priority in ongoing translational research.

Several classes of senotherapeutics have been identified (Table 1). Senolytic agents include BCL-2 family inhibitors (e.g., Navitoclax) that disrupt anti-apoptotic survival pathways (Zhu et al., 2016); tyrosine kinase inhibitors (e.g., Dasatinib) (Meiners et al., 2024; Darmawan et al., 2023; Justice et al., 2019); natural polyphenols (e.g., Fisetin, Quercetin) (Zhu et al., 2017; Hohmann et al., 2019); differentiation-modulating compounds (e.g., Peroxisome Proliferator-Activated Receptor gamma [PPARγ] agonists such as Fenofibrate) (Ansari et al., 2024; Jin et al., 2023; Vlachogiannis et al., 2023; Nogueira-Recalde et al., 2019); and cardiac glycosides that disrupt ion homeostasis by targeting the Na+/K + ATPase pump (e.g., Digoxin) (Triana-Martínez et al., 2019). Among these, BCL-2 inhibitors and Dasatinib have demonstrated efficacy against senescent fibroblasts in lung fibrosis models by overcoming their apoptotic resistance (Zhu et al., 2016; Power et al., 2023; Park and Shin, 2022). However, translating these promising findings to patients has been more difficult. A Phase-1, open-label, clinical trial of Dasatinib in combination with Quercetin (D + Q) in IPF patients reported modest improvements in physical function but did not detect changes in pulmonary function or fibrosis burden (Justice et al., 2019). The lack of pulmonary improvement despite gains in physical function suggests that systemic senolysis may benefit overall aging physiology without effectively targeting the pathogenic cells driving lung fibrosis. These results highlight the need for improved drug delivery into fibrotic tissue, and a clearer understanding of which cell populations must be eliminated to reverse fibrosis.

**TABLE 1:** Strategies to target senescence in Lung Fibrosis.

Category	Class	Example	Mechanisms/Target	Evidence level	Fibroblast-specific findings and references
Senolytics	BCL-2 family inhibitors	Navitoclax	Block anti-apoptotic signaling	*In vivo* (murine lung fibrosis models)	Clears senescent fibroblasts; reduces fibrosis; clinical use limited by thrombocytopenia. (Schafer et al., 2017; Zhu et al., 2016; Roberts et al., 2012; De Vos et al., 2021)
	Tyrosine kinase inhibitors	Dasatinib	Disrupt survival and pro-growth signaling	*In vitro* (human keloid fibroblasts); Phase I pilot (D + Q in IPF)	Overcomes apoptosis resistance in senescent fibroblasts; D + Q improved physical function but not lung function (Darmawan et al., 2023; Justice et al., 2019)
	Natural polyphenols	Fisetin, Quercetin	Multi-target stress and survival pathway inhibition	*In vivo* (murine models); *in vitro* (human IPF fibroblasts)	Quercetin enhances ligand-induced apoptosis in senescent IPF fibroblasts; reduces fibrosis *in vivo*. (Zhu et al., 2017; Hohmann et al., 2019; Ansari et al., 2024; Jin et al., 2023; Vlachogiannis et al., 2023)
	Differentiation modulators	Fenofibrate	PPARγ agonists	*In vitro* (Human chondrocytes); limited lung data	Limited direct data in lung fibroblasts; mechanism suggests potential utility (Ansari et al., 2024; Nogueira-Recalde et al., 2019)
	Cardiac glycosides	Digoxin	Disrupt Na+/K + ATPase pump	*In vitro* (chemotherapy- and bleomycin-induced senescence)	Decreases viability of senescent cells; lung fibroblast-specific data limited (Triana-Martínez et al., 2019)
Senomorphics	mTOR inhibitors	Rapamycin	Reduce mTOR complex1 (mTORC1) activation; Reduce STAT3 phosphorylation	*In vitro* (senescent fibroblasts)	Decreases SASP and β-gal expression in fibroblasts; *does not reduce* p16 expression (Demidenko et al., 2009; Wang et al., 2017)
	JAK/STAT inhibitors	Ruxolitinib	Block JAK tyrosine kinase catalytic site	*In vivo* (aged mice); limited lung fibrosis-specific data	Reduces SASP and age-related frailty in mice; direct lung fibroblast data emerging (Xu et al., 2015; Ostojic et al., 2011)
	IKK/NF-κB inhibitors	Metformin, others	Prevent NF-κB translocation to nucleus (Metformin)	*In vitro* (senescent fibroblasts)	Inhibits expression of SASP inflammatory cytokines (Moiseeva et al., 2013)
	Antioxidants	NAC, others	Reduce oxidative stress-driven SASP	*In vitro*; limited *in vivo* fibrosis data	Attenuates oxidative stress and senescence markers in fibroblasts (Ebrahimirad et al., 2025)
Emerging/Experimental	BET inhibitors	JQ1, OTX015	Epigenetic suppression of SASP or fibrotic genes transcription	*In vivo* (aged murine lung fibrosis model) and *in vitro* (fibroblast model)	Broad inhibition of pro-fibrotic cytokines and markers; accelerates fibrosis resolution in aged mice (Sanders et al., 2020; Khosla, 2023; Wakita et al., 2020; Go et al., 2021)
	cGAS–STING inhibitors	RU.521	Limit DNA-induced interferon activation	*In vitro* (alveolar epithelial cells); limited fibroblast data	Reduces IL-6 production and senescence in epithelial cells; fibroblast-specific effects under investigation (Zhang et al., 2023a; Schuliga et al., 2021b)
	p38 MAPK inhibitors	SB203580	Destabilize SASP mRNA, disrupt the signaling loop	*In vitro* (senescent lung fibroblasts)	Blocks sustained p38 signaling in senescent lung fibroblasts; targets pro-fibrotic secretome (Zhu et al., 2026; Ya and Bayraktutan, 2024; Freund et al., 2011; Alipour-Khezri et al., 2025)
Combination Strategies	Sequential senomorphic → senolytic	Multi-agent regimens	SASP suppression followed by senescent cell clearance	Conceptual/preclinical inference; direct lung fibrosis data needed	May maximize efficacy and minimize toxicity; direct lung fibrosis data needed (Childs et al., 2015; Saliev and Singh, 2025; Mansfield et al., 2024)

Alongside senolytic strategies that aim to clear senescent cells, senomorphics that dampen the SASP without eliminating the cells have been developed in parallel. Senomorphics include inhibitors of mTOR, JAK/STAT, Ikappa B Kinase (IKK)/NF-κB, and antioxidants that reduce inflammatory and oxidative stress pathways (Zhang L. et al., 2023). mTOR and JAK inhibitors are particularly relevant for fibroblasts, as they target key signaling nodes that sustain the profibrotic SASP (Xu et al., 2014; Carroll et al., 2017; Xu et al., 2015; Laberge et al., 2015; Xu et al., 2016). While senolytics offer the potential for permanent removal of senescent cells, they carry a risk of off-target toxicity due to unintended apoptosis of ‘healthy’ cells. In contrast, senomorphics may be safer for chronic use but allow senescent cells to persist (Niedernhofer and Robbins, 2018). In IPF, a combined or sequential therapeutic approach may prove optimal. For instance, suppressing inflammation with senomorphics before senolytic clearance. Combination strategies are increasingly explored in IPF, as evidenced by ongoing trials of combined antifibrotic therapies, and this rationale extends logically to senotherapeutics given the interconnected nature of senescence pathways and the heterogeneity of senescent cell populations. However, direct experimental evidence for sequential senomorphic-senolytic regimens in lung fibrosis models is currently lacking and represents an important direction for future pre-clinical work (Saliev and Singh, 2025).

Beyond currently tested senotherapeutics, additional drug classes show promising results based on mechanisms regulating the profibrotic SASP. Bromodomain and Extra-Terminal domain (BET) protein inhibitors target epigenetic regulators of SASP transcription and/or broadly suppress pro-fibrotic cytokine or markers production, and improved progressive lung fibrosis in aged mice (Sanders et al., 2020; Khosla, 2023; Wakita et al., 2020; Go et al., 2021). cGAS-STING pathway inhibitors may block the upstream SASP activation in response to cytosolic DNA, a common feature of senescent cells (Zhang J. et al., 2023; Schuliga et al., 2021b). p38 MAPK inhibitors represent other potential strategies (Zhu et al., 2026; Ya and Bayraktutan, 2024; Freund et al., 2011; Alipour-Khezri et al., 2025). In a recent report, p38 MAPK inhibitor blocks the sustained signaling of p38 MAPK in senescent lung fibroblasts, which would break the activation loop in fibroblasts (Zhu et al., 2026). While these classes have not been extensively tested in senescent fibroblasts *in vivo*, their mechanisms align closely with key drivers of the fibrotic signaling make them strong candidates for future investigation.

## 6 Discussion and future directions

Senescent fibroblasts are central drivers of lung fibrosis in the aging lung, undermining normal wound repair and perpetuating self-sustaining feedback loops that lock the tissue into progressive scarring. Targeting these cells offers a direct strategy to mitigate fibrosis, either by clearing senescent cells with senolytics or by suppressing their secretory program with senomorphics. Still, translating these insights into effective therapies faces several hurdles.

One major obstacle is the heterogeneity of senescent cell populations. SASP composition, surface marker expression, and senolytic susceptibility all vary, which depend on cell type, the initiating stressor, and the local tissue environment (Cohn et al., 2023; Ogrodnik et al., 2024). Studies to apply single-cell profiling of IPF lung tissue to define fibroblast-specific senescent subtypes and pinpoint which populations are most therapeutically relevant would be helpful to clear the obstacle. Early work in this area is already uncovering new senescence signatures and potential disease targets (Habermann et al., 2020; Hughes et al., 2025). One such study (Hughes et al., 2025) combined single-cell RNA sequencing of IPF lung tissue with weighted gene co-expression network analysis to identify senescence-associated gene signatures in primary lung fibroblasts. This work uncovered a novel cluster of 163 genes enriched in senescent fibroblasts (Hughes et al., 2025). These signatures still require validation in larger datasets, but the work highlights the potential of single-cell approaches to uncover new biomarkers and therapeutic targets in IPF.

Another challenge is the optimal delivery and treatment timing of senotherapeutics. Systemic delivery of senolytics raises concerns about off-target effects. Navitoclax, for example, causes dose-limiting thrombocytopenia and neutropenia (Roberts et al., 2012; De Vos et al., 2021). Inhaled formulations could help concentrate these drugs in the lung while limiting systemic exposure. Other approaches, like nanoparticle-based carriers or antibody-drug conjugates directed at senescence surface proteins, are predicted to further improve specificity (Shi et al., 2024; Takaya et al., 2023).

In addition, dosing regimens and treatment sequencing add another layer of complexity. Intermittent dosing is generally preferred to preserve the beneficial roles of transient senescence in wound healing and tumor suppression, though the best schedule likely varies by agent and disease stage (Justice et al., 2019; Lelarge et al., 2024). Combination strategies also deserve attention. For instance, suppressing SASP-driven inflammation with senomorphics might make lingering senescent fibroblasts more susceptible to later senolytic clearance. Direct evidence for this kind of sequential approach in lung fibrosis models is still lacking, but the concept is worth exploring.

Furthermore, another consideration is to determine which IPF patients stand to benefit from a given senotherapeutic. In addition to the heterogeneity of senescent cell populations, IPF itself is a highly variable disease with an unpredictable clinical course (Cohn et al., 2023; Ogrodnik et al., 2024; Clarke et al., 2017). Together, this means patient stratification will likely be needed to identify those most likely to respond. Such stratification requires reliable non-invasive biomarkers of pulmonary senescence, which are not currently available. Circulating factors, such as senescence-regulatory factors, have been explored as potential markers in age-related disease, but none have yet been validated for clinical use in IPF (Maroun et al., 2024; Cummings et al., 2024). Future trials that incorporate exploratory biomarker endpoints may help lay the groundwork for more personalized treatment strategies down the line. A deeper understanding of fibroblast-specific senescence programs will be central to these efforts. Ultimately, progress in defining senescent fibroblast biology, improving targeted delivery, and identifying which patients stand to benefit will determine whether senotherapies can move from promising concept to meaningful clinical reality for those affected by age-associated fibrotic lung disease.
